# Grading retinal displacement after retinal detachment repair: a prospective study linking displacement grade and visual outcome

**DOI:** 10.1007/s00417-025-06897-4

**Published:** 2025-07-29

**Authors:** Ofri Vorobichik Berar, Gabriel Katz, Miri Meira Fogel Levin, Daniel David, Gal Yaakov Cohen, Lital Smadar, Rachel Shemesh, Eva Platner, Orit Vidne-Hay, Amir Alhallel, Avner Hostovsky

**Affiliations:** 1 Goldschleger Eye Institute, Sheba Medical Center, Tel-Hashomer, Israel; 2 Affiliated to the Sackler Faculty of Medicine, Tel Aviv University, Tel Aviv, Israel

**Keywords:** Retinal detachment, Retinal displacement, Visual outcome

## Abstract

**Background:**

To evaluate the impact of surgical technique, preoperative findings, and the degree of retinal displacement on visual outcomes in patients undergoing repair for rhegmatogenous retinal detachment (RRD).

**Methods:**

In this prospective cohort study, all consecutive patients with RRD treated at Sheba Medical Center between March 2021 and March 2022 were included. Data on surgical techniques, visual acuity (VA), preoperative posterior vitreous detachment (PVD), and postoperative imaging were collected. Retinal displacement was graded based on fundus autofluorescence and correlated with visual outcomes.

**Results:**

A total of 128 eyes from 128 patients (mean age 56.7 ± 15.4 years) were analyzed. Pars plana vitrectomy (PPV) was the most common procedure (79.9%), followed by scleral buckle (SB, 18.8%) and pneumatic retinopexy (2.3%). Significant improvement in VA was observed across all surgical groups. Retinal displacement was more frequent following PPV (68%) compared to SB (16%). Higher displacement grades (2–3) were associated with significantly worse final VA (*p* = 0.024). The presence of preoperative PVD positively correlated with surgical success in the PPV group (*p* = 0.003). Early postoperative signs of displacement included retinal folds and photoreceptor changes.

**Conclusions:**

Preoperative PVD status is a significant predictor of surgical success in PPV-treated RRD. Grading of retinal displacement offers a practical tool to assess its functional impact, with higher grades correlating with worse visual outcomes. Identifying risk factors for visually significant displacement may improve surgical decision-making and patient prognosis.

## Key messages

*****What is known***:**

- Retinal displacement is a known postoperative finding in patients undergoing pars plana vitrectomy (PPV) for rhegmatogenous retinal detachment (RRD).
- Scleral buckle surgery is associated with lower rates of retinal displacement compared to vitrectomy.

*****What is new***:**

- A novel grading system for retinal displacement, based on vascular shift in the vertical foveal line, was introduced and shown to correlate with visual acuity outcomes.
- Preoperative posterior vitreous detachment (PVD) status significantly impacts surgical success, particularly in PPV cases.
- High-grade retinal displacement (grades 2–3) is associated with worse visual acuity following RRD repair.

## Introduction

Retinal detachment is defined as the separation of the neurosensory retina from the underlying retinal pigment epithelium. Among its various forms, rhegmatogenous retinal detachment (RRD) is the most common [1]. Although retinal detachment can occur at any age, its incidence peaks in the seventh to eighth decades of life, likely due to posterior vitreous detachment (PVD). The reported incidence of RRD varies across populations and studies, ranging between 6 and 18 cases per 100,000 individuals per year [2–6].

Surgical intervention is the mainstay of treatment for RRD. Techniques include scleral buckle (SB), pneumatic retinopexy, and pars plana vitrectomy (PPV) with either gas or silicone oil tamponade. While anatomical success rates for these procedures are generally high—recently reported at 93.1% for SB and 91.8% for PPV [7]. The success rate for Phaco-vitrectomy for primary RRD is about 85% [8].

Anatomical reattachment does not always translate to favorable visual outcomes. Visual prognosis depends not only on surgical success but also on preoperative visual acuity and the macular status at presentation. Factors such as duration of detachment and macular elevation height also play important roles [9–11]. 

In the past decade, increasing attention has been given to retinal displacement as a postoperative finding following RRD repair. Studies have shown that the prevalence of displacement varies depending on the surgical method. For example, Lee et al. reported postoperative macular displacement in 72% of patients undergoing PPV with gas tamponade, while no displacement was observed in patients treated with SB [12] A recent meta-analysis confirmed significantly higher rates of displacement following PPV compared to SB [13].

Although retinal displacement has been associated with visual distortion and metamorphopsia [12] its effect on postoperative best-corrected visual acuity (BCVA) remains inconclusive. Some studies report no significant difference in visual acuity between patients with and without displacement [14–16]. while others suggest a potential impact [17]. n this study, we aimed to evaluate the correlation between surgical technique, preoperative findings (including PVD status), and the degree of retinal displacement, and to assess their combined effect on visual outcomes following RRD repair.

## Methods

We conducted a prospective study of all consecutive patients who presented with rhegmatogenous retinal detachment (RRD) at Sheba Medical Center, Israel, between March 2021 and March 2022.

### Inclusion and exclusion criteria

All patients aged 18 years and older with primary RRD were included. Exclusion criteria were:

- Non-rhegmatogenous retinal detachment.
- Recurrent or persistent detachment.
- History of prior intraocular surgery (except for cataract extraction).
- Follow-up of less than three months postoperatively.

### Preoperative evaluation

All patients underwent a comprehensive ophthalmologic examination, including:

- Best-corrected visual acuity (BCVA).
- Intraocular pressure measurement.
- Slit-lamp biomicroscopy.
- Dilated fundus examination.
- Optical coherence tomography (OCT) using Heidelberg Spectralis (Heidelberg Engineering Inc., Franklin, MA, USA).
- Fundus autofluorescence (FAF) imaging using Heidelberg Spectralis and Clarus™ 500 (Carl Zeiss Meditec Inc., Dublin, USA).

### Postoperative follow-up

Patients were evaluated postoperatively at the following intervals: 1 day, 1 week, 1 month, 3 months, and 6 months. Follow-up assessments included BCVA, slit-lamp examination, dilated fundus examination, and repeated OCT and FAF imaging. Postoperative images were assessed by a retina specialist blinded to the surgical technique.

Demographic data, preoperative findings, intraoperative details, and postoperative outcomes were retrieved from medical records.

### Surgical techniques

#### Scleral Buckle (SB)

All SB procedures included a 360-degree limbal peritomy, traction sutures under the rectus muscles, and cryotherapy to retinal breaks. A non-drainage technique was used with encircling sponge placement.

#### Pars Plana Vitrectomy (PPV)

Vitrectomy was performed using a 25-gauge, three-port system. Subretinal fluid was drained either through the primary retinal break using a fluid-air exchange or with the assistance of perfluorocarbon liquid (PFC), at the surgeon’s discretion. No posterior retinotomy was performed in any case. Internal tamponade was achieved with either 15% perfluoropropane (C3F8) or silicone oil, based on clinical considerations.

### Assessment of retinal displacement

Retinal displacement was evaluated using ultra-widefield fundus autofluorescence (FAF) imaging. Both CLARUS (Carl Zeiss Meditec) and Heidelberg Spectralis systems were used to obtain high-quality FAF images. Imaging was initiated approximately two weeks after surgery, with follow-up images acquired at 2 weeks,3 months, and 6 months postoperatively. All images were reviewed for the presence of retinal vessel printings (RVPs) or other signs of retinal displacement.

A sub analysis was performed to assess the correlation between the surgical technique, subretinal fluid drained methods and the tamponade use to the retinal displacement rates.

To define visually significant vascular retinal displacement, we decided to grade the vascular retinal displacement. We measured the amount of displacement from the inferior or superior retinal vein, at the vertical line across the fovea. To avoid measurements difference between OCT and FAF machines the displacement was graded clinically according to the relationship between the ghost (the hyperreflective line) and the current blood vessel. This method was consistent and repeatable.

**Fig. 1:**
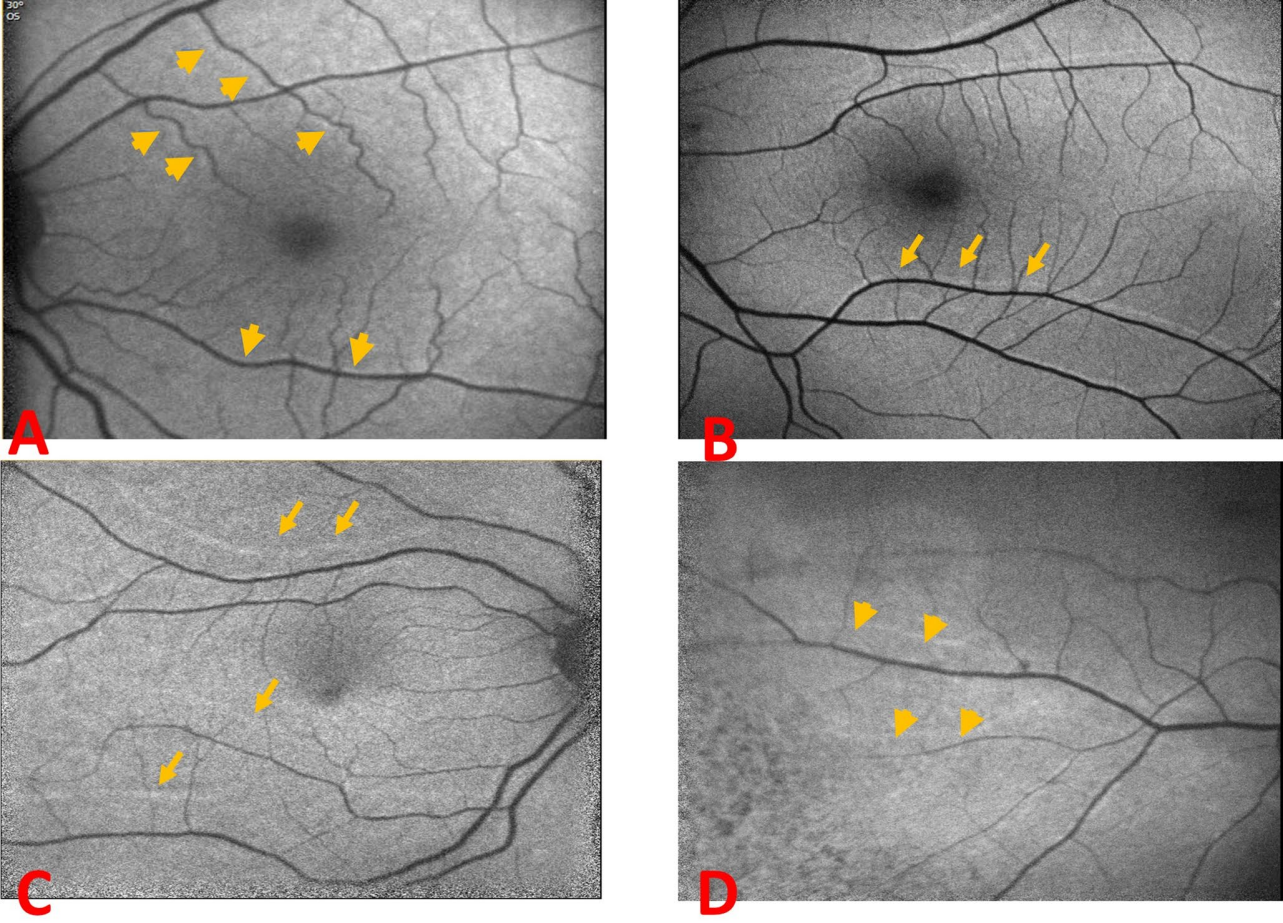
Vascular displacement grading: Grade1 shadow of retinal vessel, with no 2 separate lines (**A**), Grade 2: two separate lines (**B**). Grade 3: vessel displacement larger than the size of 2 blood vessels (**C**). Vascular displacement outside the foveal vertical line (**D**)

Grade 1 retinal displacement was defined as a shadow of hyper refractive retinal vessel attached to the current vessel, with no 2 separate lines (Fig. 1A), grade 2 vascular displacement was defined when a clear space was visible between the vessels, and they appeared as two separate lines (Fig. 1B). Grade 3 was defined as vessel displacement larger than the diameter of 2 blood vessels (Fig. 1C). Vascular displacement outside the foveal vertical line was not graded (Fig. 1D).

### Statistical analysis

Quantitative variables were described using mean, standard deviation (SD), and range. Categorical variables were expressed as frequencies and percentages. A paired analysis was used to compare pre- and postoperative BCVA. The subgroup analysis was conducted based on surgical technique.

Multivariate analysis was used to identify predictors of final visual outcome. Retinal displacement analysis was limited to macula-off cases treated with PPV and with successful initial reattachment.

All statistical analyses were performed using SPSS software, version 25.0 (IBM Corp., Armonk, NY, USA).

## Results

One hundred and twenty-eight patients underwent retinal detachment surgery. Mean age at diagnosis was 56.7 ± 16 years (range, 19–83). Demographic and clinical characteristics are listed in Table 1. Mean follow-up time was 7.5 ± 3.7 months (range, 1–17).

**Table 1:** Demographic and clinical characteristics

Gender	Male	80 (62.5%)
	Female	48 (37.5%)
VA loss (mean, days)		9 ± 3 (range, 0–60)
Time from diagnosis to surgery (mean, days)		2.6 ± 2.5 (range, 0–15)
Time from symptoms to surgery (mean, days)		11.4 ± 15 (range, 2–63)
Pre-op VA (mean, logMar)		0.8 ± 0.8
Pre-op IOP (mean, mmHg)		14.5 ± 3.5
Pre op lens status	Phakic	76 (59.4%)
	Pseudophakic	52 (40.6%)

Twenty-four patients (19%) underwent scleral buckle (SB), 86 Patients (67%) underwent PPV (± cataract), 15 (11.7%) had Band + PPV and 3 (2.3%) had pneumatic retinopexy. The most frequent tamponade was C3F8 that was used in 77 patients. Only 6 patients (4.7%) had silicone oil tamponade. PFC was used in 73 out of 101 eyes that underwent PPV.

As expected, there was a significant clinical characteristic difference between patients referred to scleral buckle and PPV (with or without band), and therefore we did not compare their clinical outcomes. Patients referred to SB were younger (34.3 years vs. 60.2, *p* < 0.001), had better visual acuity at presentation, had clear lens and did not have PVD prior to surgery (22% vs. 83%, *p* < 0.001). The clinical characteristics of the two groups are summaries in Table 2.

**Table 2:** Preoperative characteristics

	Buckle	PPV	*P* value
Age	34.3 ± 0.2	60.2 ± 0.8	*P* < 0.001
Females	66%	50%	*P* = 0.008
Visual acuity	0.28	0.89	*P* < 0.001
Macula on	33%	19%	*P* = 0.371
Clear lens	100%	26%	
PVD status	22%	83%	*P* < 0.001
Symptoms duration (days)	17.0	7.8	*P* = 0.137
Pre-op ERM	0	11%	
Pre-op MH	0	2.3%	

In each group (vitrectomy and scleral buckle) we separately performed a correlation analysis between preoperative and intraoperative variables to the final visual acuity, surgical success and vascular retinal displacement (VRD). The parameters included were age and gender, lens status, macular status (On or off), macular height during detachment, detachment location by retinal quarters, PVD status and location and number of tears.

### Success of first surgery

In the vitrectomy group, PVD status was the only preoperative variable that was correlated to the success of first surgery (*R* = 0.337, *p* = 0.003). While patients with PVD had an 89% success rate, in patients with no PVD the success rates dropped to 50%.

In the buckle group SOFS did not correlate with PVD and the only correlation that was found was to the number of tears (*R*=−0.442, *p* = 0.031).

### Retinal displacement

Ninety-seven patients (75%) had macula off RD. Of them, 76 had success in the first surgery and had imaging that was sufficient to determine presence of retinal displacement. Sixty-three of these patients underwent PPV and 13 patients underwent SB. Forty-three (68.3%) of the patients in the vitrectomy group and 2 (16%) in the SB had retinal vascular displacement. 20 patients in the vitrectomy group were categorized as having “no displacement”. No correlation was found between the present of vascular retinal displacement and fovea height (*p* = 0.353), PFC use (*p* = 0.99), type of tamponade (Gas or silicon, *p* = 0.531) detachment position by quarters (*p* = 0.617), immediate down position after surgery (*p* = 0.123) and the final visual acuity (*p* = 0.236).

There was a large variety in the displacement size of patients.

In patients with macula-off PPV with SOFS, sixteen had grade 1 retinal displacement, twenty patients had grade 2 retinal displacement and 11 had grade 3. There was statistically significant difference in final visual acuity between groups (*p* = 0.024) and the final visual acuity was worst in grade 2 and 3 vascular retinal displacement (Fig. 3).

### Postoperative OCT findings

#### Retinal fold

**Fig. 2:**
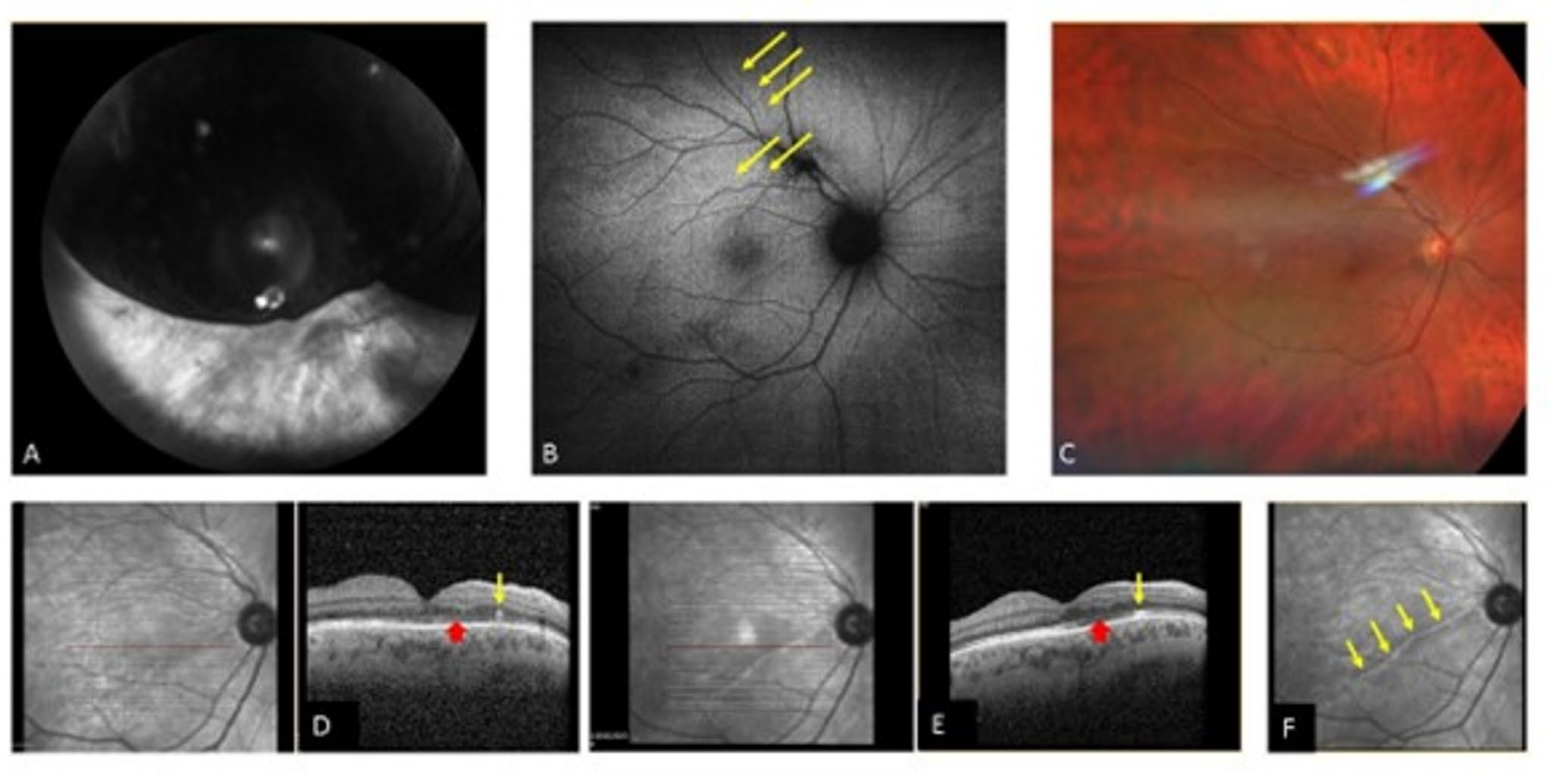
Multimodal imaging of a patient presented with superior retinal detachment. Symptoms started two days prior to arrival. **A** Wide field red free image at presentation with superior bolus retinal detachment and visual acuity of CF. **B** Autofluorescence (AF) two months after PPV with C3F8, a superior retinal vascular displacement at the area of detachment is demonstrated, involving the macula (yellow arrows). **C** Wide field color image three months after the surgery with almost normal appearance fondus, the visual acuity is 6/30. **D** OCT one month after the surgery demonstrate a retinal fold (yellow arrow) and changes in the photoreceptors in the fovea (red arrowhead). **E** OCT six months after the surgery with absorption of the retinal fold (yellow arrow) and consistent photoreceptors changes (red arrowhead). **F** Red free image twelve months after the surgery, although the retinal fold was absorbed there is still a clear hyperreflective line at the fold location (yellow arrows)

As small retinal fold is seen mainly in the first weeks after retinal detachment surgery and might not be appearance in the OCT later, an effort was made to performed and OCT in the first few weeks after the PPV. Out of 21 patients that had OCT of the macula in the first three weeks 17 (80.9%) had evidence of retinal fold in OCT (Fig. 2). All 17 patients demonstrate vascular retinal displacement in follow up pictures.

#### Photoreceptors changes and abnormal foveal reflex

**Fig. 3:**
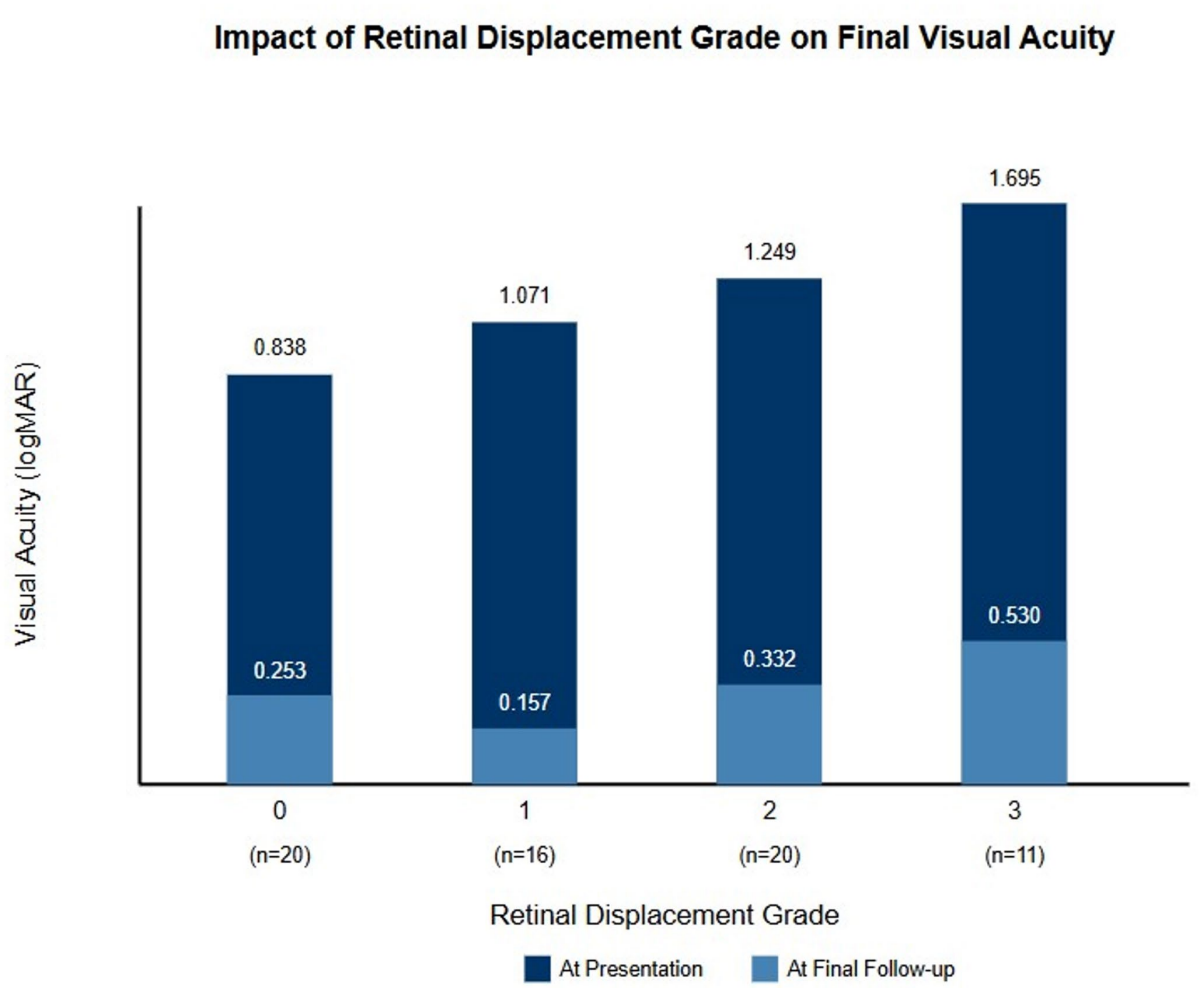
Visual acuity at presentation and at the Last follow up in patients with no, grade 1, 2 and 3 retinal vascular displacement

Seventy patients (63.6%) had photoreceptor changes after the operation (Fig. 3) and 60 patients had abnormal foveal reflex. These OCT findings were significantly more common in patients that had documented vascular retinal displacement (*p* = 0.026, *p* = 0.039 respectively).

When compared to the AF image the retinal shift and outer retinal changes are only demonstrated above the retinal fold.

## Discussion

This prospective study of 128 patients undergoing primary retinal detachment (RD) repair aimed to identify preoperative and intraoperative factors influencing anatomical and functional outcomes, with a particular focus on retinal displacement and visual acuity. Our findings reinforce previously known associations and introduce a novel, practical method to grade vascular retinal displacement.

A noteworthy finding in this study was the strong correlation between preoperative posterior vitreous detachment (PVD) and the success of the initial vitrectomy. Eyes with pre-existing PVD had an 89% success rate, while success dropped to 50% in those without PVD, although a PVD induction was performed in all of those cases This observation aligns with prior studies that found mixed outcomes based on PVD status [18, 19]. Inducing PVD intraoperatively in eyes with retinal detachment is challenging and may contribute to residual vitreous traction, increasing the risk of postoperative proliferative vitreoretinopathy (PVR). Our data suggest that in such cases, scleral buckling or combined SB-PPV may be preferable. Notably, we did not observe this complication in eyes treated with SB alone, suggesting that PPV itself may increase the risk of postoperative complications in non-PVD cases.

SB was primarily used in younger, phakic patients without PVD, reflecting standard practice. Our anatomical success rate in this group matches previous reports [20, 21] We also found that the number of retinal breaks was a negative predictor of primary success with SB, likely due to technical challenges in matching buckle placement to multiple or peripheral breaks.

Vascular retinal displacement and its influence on functional results is being researched since 2010 [22], a systematic review was recently published [13], assessing the proportion of patients with retinal displacement following retinal detachment repair. In patients that underwent PPV with gas, they reported displacement rates of 6.4–62.8% but different surgical procedures are included (especially different positioning after surgery). With face-down positioning, similar to that performed in our research, retinal displacement is described in 28–62% [15, 16, 22] of the patients. We found displacement rates of 68% in the vitrectomy group. We believe, according to recent studies, such as in roditi et al. [23], that this is an underestimation of actual displacement rate and the sub group that had a grade 0 retinal vascular displacement is a mix of patients with minimal displacement that we were not able to demonstrate and patients with displacement that was not demonstrated due to media or other retinal finding. This can explain the mixed visual acuity outcome in this group.

In the scleral buckle group we found low rates of displacement, but higher than previously described [12, 24], we believe that this difference is due to early and repeated post-surgical imaging, that detects even low grade displacement.

Other OCT findings are related to the vascular displacement, such as retinal folds, abnormal foveal reflex, and photoreceptor changes. We believe that retinal folds after the detachment are an early sign of displacement. Faravash et al. [25] pustulated that the displacement is related to the RPE re-attachment in the state of stretch or compression, retinal folds might be a sign for this compression.

Similar to prior studies, such as dell’Omo et al. [16], Cobos et al. [15] and Chelazzi et al. [14] we found no difference on visual acuity between patients with and without displacement, when no grading of displacement was performed. In an attempt to understand the effect of retinal displacement on visual acuity Casswell et al. [26] graded retinal displacement according to 2 measures: (1) the number of quadrants (around the fovea) affected, and (2) the mean amplitude of displacement. They found in post-hoc analysis that high grade displacement correlated with more distortion and worse visual acuity. Their primary outcome was to assess the head positioning effect of displacement, and they did not find correlation of positioning to final visual outcome.

Our study is the first, to our knowledge, to assess the displacement size in the vertical line in the fovea, and its influence on visual acuity and not only metamorphopsia or aniseikonia. We looked for a method that can be done in different imaging modalities, can be repeatable in different studies and may accurately reflect the amount of displacement. We showed that patients with larger retinal displacement, grade 2 and 3, had worse visual outcome. We failed to find a correlation between the retinal displacement size to clinical or surgical factors. We believe that due to the relatively small number of patients in each group our study did not have the power to find those risk factors. Our method of assessing visually significant displacement is not operator dependent and is relatively easy to repeat. We hope that the visually significant displacement found in our study, defined as Grade 2 or 3 displacement in the vertical line in the fovea will serve as an outcome measure in future studies. Further research is needed to find the surgical risk factors for visually significant retinal displacement. If we can find surgical risk factors for major displacement, we might be able to improve visual acuity in patients with RRD.

The main limitation of our study is the relatively small sample size in the high-grade displacement group. As such, we may have lacked statistical power to identify all surgical factors influencing major displacement. Further multicenter studies with larger cohorts are needed to validate our grading scale and explore its utility as a surrogate outcome measure.

## Conclusion

This study highlights the importance of preoperative PVD status in predicting surgical success following PPV and introduces a novel, clinically meaningful method for grading retinal displacement. High-grade displacement correlates with worse visual outcomes, reinforcing the need for early detection and consideration of surgical strategies that may mitigate its occurrence.
